# Abnormalities of confidence in psychiatry: an overview and future perspectives

**DOI:** 10.1038/s41398-019-0602-7

**Published:** 2019-10-21

**Authors:** Monja Hoven, Maël Lebreton, Jan B. Engelmann, Damiaan Denys, Judy Luigjes, Ruth J. van Holst

**Affiliations:** 1 Department of Psychiatry, Amsterdam UMC, University of Amsterdam, Amsterdam, The Netherlands; 2 Swiss Center for Affective Science (CISA), University of Geneva (UNIGE), Geneva, Switzerland; 3 Neurology and Imaging of Cognition (LabNIC), Department of Basic Neurosciences, University of Geneva (UNIGE), Geneva, Switzerland; 4 CREED, Amsterdam School of Economics (ASE), University of Amsterdam, Amsterdam, The Netherlands; 5 Amsterdam Brain and Cognition (ABC), University of Amsterdam, Amsterdam, The Netherlands; 6 The Tinbergen Institute, Amsterdam, The Netherlands; 7 Neuromodulation & Behavior, Netherlands Institute for Neuroscience, KNAW, Amsterdam, The Netherlands

**Keywords:** Human behaviour, Psychiatric disorders

## Abstract

Our behavior is constantly accompanied by a sense of confidence and its’ precision is critical for adequate adaptation and survival. Importantly, abnormal confidence judgments that do not reflect reality may play a crucial role in pathological decision-making typically seen in psychiatric disorders. In this review, we propose abnormalities of confidence as a new model of interpreting psychiatric symptoms. We hypothesize a dysfunction of confidence at the root of psychiatric symptoms either expressed subclinically in the general population or clinically in the patient population. Our review reveals a robust association between confidence abnormalities and psychiatric symptomatology. Confidence abnormalities are present in subclinical/prodromal phases of psychiatric disorders, show a positive relationship with symptom severity, and appear to normalize after recovery. In the reviewed literature, the strongest evidence was found for a decline in confidence in (sub)clinical OCD, and for a decrease in confidence discrimination in (sub)clinical schizophrenia. We found suggestive evidence for increased/decreased confidence in addiction and depression/anxiety, respectively. Confidence abnormalities may help to understand underlying psychopathological substrates across disorders, and should thus be considered transdiagnostically. This review provides clear evidence for confidence abnormalities in different psychiatric disorders, identifies current knowledge gaps and supplies suggestions for future avenues. As such, it may guide future translational research into the underlying processes governing these abnormalities, as well as future interventions to restore them.

## Introduction

Metacognition refers to our ability to think about, reflect, and comment upon our own thinking. Confidence judgment is one such metacognitive operation, and is described as the subjective feeling of being correct about a choice, decision or statement^1^. Not only is this feeling of confidence critical to re-evaluate previous decisions, it can also guide future decision-making and drive reasoning and social interactions^2^. Producing accurate confidence judgments is an individual ability, which seems stable across different sensory modalities^3–6^, time-points^7^, and across cognitive domains^8^ (but see^9,10^).

The hypothesis that inaccurate confidence judgments can lead to detrimental decision-making—bearing extensive negative consequences for society and the individual—is supported by both theoretical and experimental consensus^11–13^. Systematically inaccurate confidence judgments could contribute to persistent pathological decision-making observed in psychiatric disorders. For example, underconfidence in memory may result in compulsory checking behavior as observed in patients suffering from obsessive-compulsive disorder (OCD). On the other hand, overconfidence in erroneous beliefs could underpin delusional thinking as observed in schizophrenia patients. Yet, to date an overview of abnormalities in confidence judgments across psychiatric disorders is lacking.

Here, we review studies of confidence in subclinical and clinical psychiatric populations to apprehend the associations between confidence abnormalities and psychiatric disorders. Our review focuses on OCD, schizophrenia, addiction, anxiety, and depression, and includes studies in both subclinical and clinical populations. This is because psychiatric disorders have been proposed to be characterized by both qualitative and quantitative shifts in behavior^14^, which can be represented by the visible part of a continuum of symptom severity, the lower end of which would be subclinical^15–18^. Finally, we discuss the benefits of transdiagnostic approaches to investigate confidence and psychiatric symptoms in the general population. Insight into confidence abnormalities could reveal new targets for early interventions. Overall, this review provides a comprehensive framework for the investigation of confidence in psychiatry. It also highlights the methodological challenges and limitations present in this line of research, and delineates suggestions for future avenues of research. Targeting confidence abnormalities in psychiatry could help alleviate symptoms and improve treatment outcomes.

## Methods

Two separate systematic literature searches for subclinical and clinical populations were conducted through the electronic database PubMed in October 2018, using the following key terms:

(1) (“confiden*” OR “metacogniti*” OR “meta-cogniti*”) AND (“psychiatr*” OR “impulsiv”* OR “complusiv*” OR “transdiagnostic**” OR “trans-diagnostic*” OR “individual differences” OR “symptom*” OR “healthy”). (862 hits)

(2) (“confiden*” OR “metacogniti*” OR “meta-cogniti*”) AND (“depressi*” OR “schizophr*” OR “obsessive compulsive*” OR “OCD” OR “obsessive-compulsive” OR “addict*” OR “substance*” OR “psychiatr*” OR “eating” OR “MDD” OR “gambl*” OR “anxiety*”). (811 hits)

The search was not limited regarding year of publication. We chose not to include autism spectrum disorder (ASD) and attention-deficit hyperactivity disorder (ADHD) for reasons of clarity. Exclusion criteria were non-English manuscripts; studies using questionnaires to assess confidence, and clinical trials assessing effectiveness of metacognitive therapy. All duplicates were removed, abstracts were screened and full texts of relevant studies were reviewed. From the reference lists of selected papers, additional studies and relevant reviews or meta-analyses were included.

## Results

**Fig. 1:**
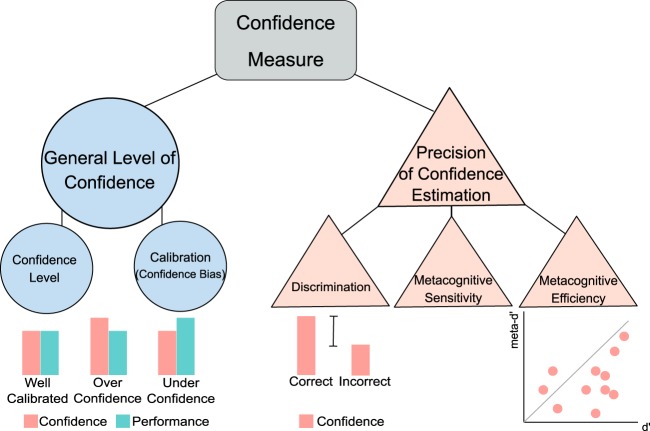
Measures of confidence. Confidence measures can be divided into general measures of confidence level and precision measures of confidence estimation. To assess someone’s general level of confidence, confidence level or calibration can be analyzed. Calibration (or confidence bias) is usually calculated as the difference between mean task performance and confidence. This results in overconfidence when confidence levels are higher than performance levels, and underconfidence vice versa. To assess someone’s precision of confidence estimation, confidence discrimination, metacognitive sensitivity or metacognitive efficiency can be analyzed. Confidence discrimination refers to the difference in confidence levels between correct and incorrect choices. The larger this difference, the higher the discriminatory accuracy of confidence, signaling an increased ability to recognize accurate from inaccurate performance by using one’s metacognitive report. Confidence discrimination is sometimes referred to as ‘the confidence gap’. Confidence bias and discrimination are two independent aspects of metacognition: an individual might be underconfident, but still be highly sensitive to discriminate between accurate and inaccurate performance with their confidence. Similar to discrimination, metacognitive sensitivity, also referred to as parameter *meta-d’*, aims to measure the ability of a metacognitive observer to discriminate between correct and incorrect trials with their confidence judgments. Yet, it uses a more sophisticated calculation that is bias free, and controls for performance confounds. On the other hand, metacognitive efficiency, referred to as meta-*d’*/*d’*, indicates how well perceptual information (*d’*) is used to form a metacognitive report (*meta-d’)*. When meta-*d’*/*d’*, or the *M-ratio*, equals 1 (i.e. indicated by the line in the graph), this signals a metacognitively ideal observer that uses all perceptual information captured in *d’* for the formation of a metacognitive report. When meta-*d*’/*d’* < 1, not all information was used to form a metacognitive report, corresponding to lower metacognitive efficiency. When meta-*d’*/*d’* > 1, the observer retrieved additional information to form a metacognitive report, corresponding to higher metacognitive efficiency

**Fig. 2:**
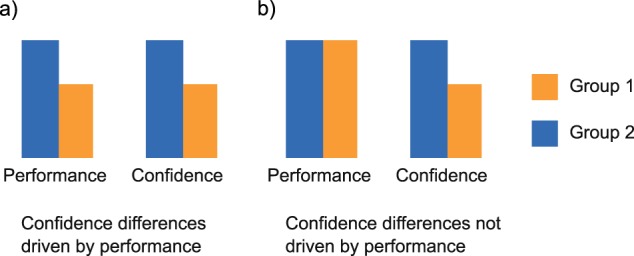
Confidence differences confounded by intergroup differences in first-order performance. **a** The difference in first-order performance between groups might result in untrue differences of confidence between groups. **b** First-order performance is equal between groups and therefore specific effects of group identity on confidence are isolated. This figure illustrates the need for bias free measures, such as meta-d’ and metacognitive efficiency, which control for performance differences between groups

We identified 83 studies that met inclusion criteria. Table 1 shows an overview of the task domains, the metacognitive measures and the most commonly used paradigms in these studies. Briefly, three types of confidence measures are often evaluated. Retrospective confidence judgements assess the correctness of a choice^19^. Feeling of Knowing (FOK) and Judgments of Learning (JOL) are prospective confidence judgments about one’s ability to later retrieve knowledge about a specific subject (FOK) or about a learned cue or cue association (JOL). However, retrospective and prospective judgments are considered to be different^7,20^, since they rely on distinct cognitive resources and are influenced by separate parameters^7^, and should therefore not be used interchangeably. In the current review we mostly focus on retrospective judgments, but for the sake of completeness we also include studies using prospective judgements. Confidence accuracy measures can be derived from comparing retrospective confidence judgements to objective task performance (Fig. 1). Confidence judgments are deemed more accurate when correct choices are held with higher confidence than incorrect choices (discrimination), and when average confidence matches average performance (calibration). Yet, confidence measures can be confounded by changes in first-order performance (Fig. 2). Therefore, recently bias free measures of confidence have been developed that rest on the foundations of signal detection theory (i.e. metacognitive sensitivity, or *meta-d’*)^21–23^, which measure the ability to discriminate between correct and incorrect choices with confidence judgments while controlling for confounds. Moreover, metacognitive efficiency, or *meta-d’/d’*, measures how efficiently perceptual information is used to form a metacognitive report. For further details on confidence accuracy metrics, see Fig. 1.

**Table 1:** Most commonly studied cognitive domains, paradigms, and measures

Domain	Paradigm	Metacognitive measure	Description of paradigm
Memory	Repeated Checking Task	Confidence level (N-BF)	Participants manipulate different objects (e.g. light switches) and rate their memory confidence. The effects of repeated checking on memory confidence are assessed.
	Repeated Cleaning Task	Confidence level (N-BF)	Participants clean different objects and rate their memory confidence in cleaning those objects. The effects of repeated cleaning on memory confidence are assessed.
	Verbal Memory Task	Confidence level and FOK/JOL measures (N-BF)^a^	Participants memorize words and after a time interval perform a recall or recognition and rate their memory confidence.
	Visual Memory Task	Confidence level and FOK/JOL measures (N-BF)^a^	Participants memorize visual stimuli and after a time interval perform a recall or recognition and rate their memory confidence.
	False-Memory Task	Confidence level, confidence in errors and discrimination (N-BF)	Most studies made use of the Deese-Roediger-McDermott (DRM) paradigm. Word lists are presented and after a time interval a recognition test with old and new words (i.e. lure words) is administered and memory confidence is asked.
	Source-Monitoring Task	Confidence level, confidence in errors and discrimination (N-BF)	A wordlist is presented and participants create semantic associations for each word. Afterwards, participants recognize original (old) and self-created (new) words, their source (i.e. experimenter or self) and rate their memory confidence.
Perception	Perceptual Decision Making Task	Confidence level (N-BF), metacognitive sensitivity (i.e. meta-d’) and efficiency (i.e. meta-d’/d’) (BF)	Participants make a two-alternative decision about perceptual stimuli (i.e. which box contains most dots) and rate their confidence in each decision.
General Knowledge	General Knowledge Task	Confidence level (N-BF)	Participants answer general knowledge questions and rate their level of confidence.
Action	Muscle Tension Task	Confidence level (N-BF)	Participants produce certain levels of muscle tension and rate their confidence about their subjective muscle tension estimates.
Other	Predictive Inference Task	Confidence level (N-BF)	Participants predict the position of a certain particle and state their confidence in their prediction, while the environment is changing over time.
	Wisconsin Card Sorting Task	Confidence level (N-BF)	Participants figure out a sorting rule and rate their confidence in this rule. The sorting rule changes over time and the participants have to relearn the rule.
	Emotion Task	Confidence level (N-BF)	Participants recognize facial emotions and state their confidence.

Most tasks involve retrospective confidence judgements after every decision or action

*FOK* feeling of knowing, *JOL* judgement of learning, *N-BF* non bias free, *BF* bias free

^a^Task paradigm that uses both prospective and retrospective confidence judgments

### OCD

OCD is a psychiatric condition associated with repetitive and functionally impairing actions (i.e. compulsions, such as checking behaviors), mostly performed to alleviate distress induced by intrusive thoughts (i.e. obsessions)^24,25^.

#### Subclinical: obsessive-compulsive tendencies and compulsivity

Individuals can express compulsivity or obsessive-compulsive tendencies at varying levels of severity without receiving a diagnosis for OCD. Thirteen studies assessing the link between confidence and subclinical OCD symptoms were identified (Table 2a). Two studies found lowered confidence associated with high obsessive-compulsive (OC) tendencies^26,27^ using a false bio-feedback task in which participants evaluated their muscle tension. High OC individuals showed more reliance on false feedback and lower confidence in evaluating their muscle tension while the influence of feedback on muscle tension was similar between high and low OC groups. Other studies have not found direct differences in confidence ratings or calibration between individuals with high and low OC tendencies^28–30^, but a subset of these studies has identified other metacognitive effects. Hauser et al.^29^, used a motion detection task and found lower metacognitive efficiency (*meta-d’/d’*) in highly compulsive participants, suggesting that high OC subjects do not utilize all accessible information to form a metacognitive report. Ben Shachar et al.^28^ did not find any differences between high and low OC groups in any confidence measure they used (i.e., confidence level, calibration and discrimination) in a general knowledge task. However, they report that high OC participants were more reluctant to report their answers implicating that they required a higher level of confidence to act on their answer.

**Table 2:** Overview of reviewed studies

Authors	Year	Sample size and study populations	Task	Results	Performance bias free
**(A) Overview of subclinical OCD studies**					
Ashbaugh & Radomsky^32^	2007	152 HC	Repeated Checking Task^a^	↓ confidence high-checkers vs low-checkers	−
Ben Shachar et al.^28^	2013	47 HC; high and low OC tendencies	General Knowledge Task	== confidence high vs low OC tendencies	+
Coles, Radomsky & Horng^33^	2006	S1: 51 HC S2: 81 HC	Repeated Checking Task^a^	S1 & S2: ↓ confidence with repeated checking	+
Cuttler et al.^38^	2013	199 HC	Prospective Memory Task	↓ confidence undermined group	+
Fowle & Boschen^37^	2011	60 HC	Repeated Cleaning Task^a^	no increase in confidence for repeatedly cleaned items, increase in confidence for non-repeatedly cleaned items	+
Hauser et al.^29^	2017	40 HC; high and low OC tendencies	Global Motion Detection Task	↓ metacognitive efficiency high compulsive group	++
Lazarov et al.^26^	2012	38 HC; high and low OC tendencies	False Feedback Muscle Tension Task	↓ confidence high compulsive group	+
Radomsky, Gilchrist & Dussault^35^	2006	55 HC	Repeated Checking Task^a^	↓ confidence with repeated checking	+
Radomsky & Alcolado^34^	2010	62 HC	Repeated Mental Checking Task^a^	↓ confidence with repeated checking	−
Rouault et al.^30^	2018	S1: 498 HC S2: 497 HC	Perceptual Decision-Making Task	S1: no relationship OCD symptoms and confidence S2: no relationship OCD symptoms and confidence or metacognitive efficiency AD symptom dimension ↓ confidence and ↑ metacognitive efficiency, CIT symptom dimension ↑ confidence and ↓ metacognitive efficiency	++
Van den Hout & Kindt^31^	2003a	S1: 39 HC S2: 40 HC	Repeated Checking Task^a^	S1 and S2: ↓ confidence with repeated checking	+
Van den Hout & Kindt^36^	2003b	40 HC	Repeated Checking Task^a^	↓ confidence with repeated checking	+
Zhang et al.^27^	2017	S1: 30 HC S2: 32 HC	False Feedback Muscle Tension Task	S1 and S2: ↓ confidence high compulsive group	+
**(B) Overview of clinical OCD studies**					
Boschen & Vuksanovic^53^	2007	15 OCD, 40 HC	Repeated Checking Task^a^	↓ confidence OCD vs HC ↓ confidence with repeated checking	+
Bucarelli & Purdon^47^	2016	30 OCD, 18 anxious controls	Repeated Checking Task^a^	== confidence OCD vs anxious controls	−
Cougle, Salkovskis & Wahl^40^	2007	39 OCD checkers, 20 OCD non-checkers, 22 anxious controls, 69 HC	Memory Task	↓ confidence OCD vs HC and anxious controls	−
Dar et al.^59^	2000	20 OCD checkers, 29 PD, 23 HC	General Knowledge Task	↓ confidence OCD vs HC	+
Dar^58^	2004	S1: 20 OCD checkers, 20 PD, 20 HC S2: 15 OCD checkers, 15 HC S3: 6 OCD checkers, 6 HC	General Knowledge Task	S1, S2, and S3: ↓ confidence OCD vs both control groups S1, S2, and S3: ↓ confidence with repeated checking	+
Foa et al.^41^	1997	15 OCD, 15 HC	Memory Task	↓ confidence OCD vs HC	+
Hermans et al.^57^	2008	16 OCD, 16 clinical controls, 16 HC	Repeated Actions Task^a^	↓ confidence OCD vs both control groups	−
Karadag et al.^42^	2005	32 OCD, 31 HC	Memory Task	↓ confidence OCD vs HC	+
Lazarov et al.^60^	2014	20 OCD, 20 anxious controls, 20 HC	False Feedback Muscle Tension Task	↓ confidence OCD vs HC and anxious controls	+
Macdonald et al.^43^	1997	10 OCD checkers, 10 OCD non-checkers, 10 HC	Memory Task	↓ confidence OCD checkers vs non-checkers and HC	+
McNally & Kohlbeck^39^	1993	12 OCD checkers, 12 OCD non-checkers, 12 HC	Reality Monitoring Task	↓ confidence OCD vs HC	+
Moritz et al.^48^	2006	17 OCD checkers, 10 OCD non checkers, 51 HC	Source Memory Task	== confidence OCD vs HC	+
Moritz et al.^54^	2007	28 OCD, 28 HC	Memory Task^a^	↓ confidence OCD vs HC under high responsibility	+
Moritz et al.^49^	2009a	43 OCD, 46 HC	Memory Task	== confidence OCD vs HC	+
Moritz et al.^51^	2009b	32 OCD, 32 HC	Memory Task	== confidence OCD vs HC	+
Moritz et al.^50^	2011	30 OCD, 20 HC	Memory Task	== confidence OCD vs HC	+
Moritz & Jaeger^44^	2018	26 OCD, 21 HC	Memory Task	↓ confidence OCD vs HC	+
Radomsky, Rachman & Hammond^55^	2001	11 OCD	Repeated Checking Task^a^	↓ confidence under high responsibility	−
Tekcan, Topçuoglu & Kaya^52^	2007	25 OCD checkers, 16 OCD non-checkers, 27 HC	Memory Task	== confidence OCD vs HC	+
Tolin et al.^56^	2001	14 OCD, 14 anxious controls, 14 HC	Repeated Memory Task^a^	↓ confidence OCD vs both control groups with repetition	+
Tuna, Tekcan & Topçuoglu^46^	2005	17 OCD, 16 subclinical checkers, 15 HC	Memory Task	↓ confidence OCD vs HC	−
Vaghi et al.^61^	2017	24 OCD, 25 HC	Predictive Inference Task	== confidence OCD vs HC	**−**
Zitterl et al.^45^	2001	27 OCD, 27 HC	Memory Task	↓ confidence OCD vs HC	−
**(C) Overview of subclinical schizophrenia studies**					
Koren et al.^65^	2017	61 help seeking adolescents	Verbal Memory, Executive – and Social Functioning Tasks	Positive relationship self-disturbance and meta-cognitive control	+
Laws & Bhatt^70^	2005	105 HC	Memory Task	↑ confidence in errors high delusion-proneness ↓ discrimination high delusion-proneness	−
Mckay, Langdon & Coltheart^68^	2006	58 HC	Reasoning Task	↑ confidence high delusion-proneness	−
Moritz et al.^71^	2014	2008 HC	Visual Perception Task	↑ confidence in errors high paranoia ↓ discrimination high paranoia	−
Moritz et al.^73^	2015	2321 HC	General Knowledge Task^a^	↑ confidence in errors high paranoia, exaggerated with high competence or easy questions ↓ discrimination high paranoia, exaggerated with high competence or easy questions	−
Rouault et al.^30^	2018	S1: 498 HC S2: 497 HC	Perceptual Decision-Making Task	S1: No relationship SCZ symptoms and confidence S2: No relationship SCZ symptoms and confidence or metacognitive efficiency AD symptom dimension ↓ confidence and ↑ metacognitive efficiency, CIT symptom dimension ↑ confidence and ↓ metacognitive efficiency	++
Scheyer et al.^66^	2014	78 help seeking adolescents	Verbal memory, executive functioning and social functioning tasks	= = confidence high vs low psychosis-prone groups	+
Warman^69^	2008	70 HC	Decision-making task	↑ confidence high delusion-proneness	−
**(D) Overview of clinical schizophrenia studies**					
Bacon et al.^95^	2001	19 SCZ, 19 HC	General Knowledge Task^b^	= = confidence SCZ vs HC ↓ FOK ratings SCZ vs HC	−
Bacon & Izaute^96^	2009	21 SCZ, 21 HC	Memory Task^b^	↓ FOK ratings SCZ vs HC	−
Bhatt, Laws & McKenna^74^	2010	25 SCZ, 20 HC	False-Memory Task	↑ confidence in errors SCZ vs HC ↓ discrimination SCZ vs HC	−
Bruno et al.^93^	2012	28 SCZ, 14 HC	Emotional and Non-Emotional WCST	= = discrimination SCZ vs HC, but ↓ metacognitive performance SCZ vs HC	+
Davies et al.^92^	2018	41 FEP, 21 HC	Perceptual Decision-Making Task	↓ meta-d’ FEP vs HC	++
Eifler et al.^75^	2015	32 SCZ, 25 HC	False-memory Task	↑ confidence in errors SCZ vs HC ↓ discrimination SCZ vs HC	−
Eisenacher et al.^86^	2015	34 at risk patients, 21 FEP, 38 HC	Verbal Recognition Task	↑ confidence in errors at risk and FEP vs HC ↓ discrimination at risk and FPE vs HC	+
Gaweda, Moritz & Kokoszka^76^	2012	32 SCZ, 32 HC	Source-Monitoring Task	↑ confidence in errors SCZ vs HC ↓ discrimination SCZ vs HC	−
Gaweda et al.^87^	2018	36 at risk patients, 25 FEP, 33 HC	Source-Monitoring Task	↑ confidence in errors UHR and FEP vs HC ↓ discrimination UHR and FEP vs HC	−
Kircher et al.^77^	2007	27 SCZ, 19 HC	False-Memory Task	↑ confidence (more so in errors) SCZ vs HC	+
Köther et al.^88^	2012	76 SCZ, 30 HC	Emotion Recognition Task	↑ confidence in errors SCZ vs HC ↓ discrimination SCZ vs HC	−
Moritz & Woodward^78^	2002	23 SCZ, 15 HC	Source-Monitoring Task	↑ confidence in errors SCZ vs HC ↓ discrimination SCZ vs HC	−
Moritz, Woodward & Ruff^79^	2003	30 SCZ, 21 HC	Source-Monitoring Task	↑ confidence in errors SCZ vs HC ↓ discrimination SCZ vs HC	−
Moritz et al.^80^	2004	20 SCZ, 20 HC	False-Memory Task	↑ confidence in errors SCZ vs HC ↓ discrimination SCZ vs HC	−
Moritz et al.^81^	2005	30 SCZ, 15 HC	Source-Monitoring Task	↑ confidence in errors SCZ vs HC ↓ discrimination SCZ vs HC	−
Moritz & Woodward^84^	2006	31 SCZ, 48 psychiatric controls, 61 HC	Source-Monitoring Task	↑ confidence in errors SCZ vs both control groups ↓ discrimination SCZ vs both control groups	+
Moritz, Woodward & Rodriguez-Raecke^82^	2006	35 SCZ, 34 HC	False-Memory Task	↑ confidence in errors SCZ vs HC ↓ discrimination SCZ vs HC	−
Moritz, Woodward & Chen^85^	2006	30 FEP, 15 HC	Source-Monitoring Task	↑ confidence in errors FEP vs HC ↓ discrimination FEP vs HC	−
Moritz et al.^83^	2008	68 SCZ, 25 HC	False Visual Memory Task	↑ confidence in errors SCZ vs HC ↓ discrimination SCZ vs HC	−
Moritz et al.^89^	2012	23 SCZ, 29 HC	Emotion Perception Task	↑ confidence in errors SCZ vs HC ↓ discrimination SCZ vs HC	+
Moritz et al.^91^	2014	55 SCZ, 58 OCD, 45 HC	Perceptual Decision-Making Task	↑ confidence in errors SCZ vs HC ↓ discrimination SCZ vs HC	+
Peters et al.^94^	2007	23 SCZ, 20 HC	False-Memory Task	↑ confidence in errors HC vs SCZ ↓ discrimination SCZ vs HC	+
Peters et al.^90^	2013	27 SCZ, 24 HC	Emotional Memory Task	↑ confidence in errors SCZ vs HC ↓ discrimination SCZ vs HC	−
**(E) Overview of subclinical addiction studies**					
Goodie^100^	2005	S1: 200 HC S2: 384 HC	General Knowledge Task	S1 & S2: ↑ overconfidence problem and possible pathological gamblers	−
Lakey, Goodie & Campbell^101^	2007	221 HC	General Knowledge Task & Iowa Gambling Task	↑ overconfidence problem and possible pathological gamblers	−
Rouault et al.^30^	2018	S2: 497 HC	Perceptual Decision-Making Task	S2: No relationship alcoholism symptoms and confidence or metacognitive efficiency AD symptom dimension ↓ confidence and ↑ metacognitive efficiency, CIT symptom dimension ↑ confidence and ↓ metacognitive efficiency	++
**(F) Overview of clinical addiction studies**					
Brevers et al.^102^	2014	25 GD, 25 HC	Grammar Task	Disconnection confidence and accuracy GD	−
Le Berre et al.^103^	2010	28 AUD, 28 HC	Memory Task^b^	↑ FOK judgments AUD vs HC	−
Mintzer & Stitzer^105^	2002	18 MMP, 21 HC	Memory Task	↑ confidence for errors MMP vs HC ↓ discrimination MMP vs HC	−
Moeller et al.^104^	2016	14 remitted CUD, 8 active CUD, 13 HC	Perceptual Decision-Making Task	↓ metacognitive efficiency active CUD vs remitted CUD and HC	++
Sadeghi et al.^106^	2017	23 MMP, 24 HC	Memory & Perceptual Task	↓ metacognitive efficiency MMP vs HC perceptual task, but not memory task	++
**(G) Overview of subclinical depression/anxiety studies**					
Dunning & Story^118^	1991	S1: 164 HC S2: 259 HC	Future Prediction Task	S1 and S2: ↑ confidence depressed vs non-depressed	−
Quiles, Prouteau & Verdoux^119^	2015	50 HC	WCST, Digit Span, Memory Task and Emotion Recognition Task	No relationship confidence and depression/anxiety symptoms	−
Rouault et al.^30^	2018	S1: 498 HCS2: 497 HC	Perceptual Decision-Making Task	S1: Negative relationship confidence levels and depression/anxiety symptoms S2: Negative relationship confidence levels and anxiety symptoms, no relationship with metacognitive efficiency AD symptom dimension ↓ confidence and ↑ metacognitive efficiency. CIT symptom dimension ↑ confidence and ↓ metacognitive efficiency	++
Soderstrom, Davalos & Vásquez^117^	2011	97 HC	Memory Task^b^	↓ calibration based on JOL mildly depressed vs HC = = calibration based on JOL moderate depressed vs HC	−
Stone, Dodrill & Johnson^116^	2001	200 HC	General Knowledge Task	↓ confidence depressed group	+
**(H) Overview of clinical depression/anxiety studies**					
Bucarelli & Purdon^47^	2016	30 OCD, 18 ANX	Repeated Checking Task	== confidence ODC vs ANX	−
Dar et al.^59^	2000	20 OCD checkers, 29 PD, 23 HC	General Knowledge Task	== confidence PD vs OCD and HC	+
Fieker et al.^123^	2016	45 MDD, 30 HC	Emotional Perception Task	Negative correlation confidence and depression severity	+
Fu et al.^121^	2005	15 MDD, 15 recovered MDD patients, 22 HC	Memory, General Knowledge, Perceptual and Social Judgment Task	↓ confidence MDD vs HC == confidence recovered MDD vs HC and MDD	−
Hancock, Moffoot & O’Carroll^120^	1996	14 MDD, 14 recovered MDD patients, 14 HC	General Knowledge Task	↓ confidence for correct answers in MDD vs HC == confidence recovered MDD vs HC	+
Lazarov et al.^60^	2014	20 OCD, 20 ANX, 20 HC	False Feedback Muscle Tension Task	↓ confidence OCD vs ANX and HC	+
Szu-Ting Fu et al.^122^	2012	23 MDD, 22 dysphoria patients, 32 HC	Memory Task	↓ confidence MDD vs HC and dysphoria	−
Tolin et al.^56^	2001	14 OCD, 14 ANX, 14 HC	Memory Task	↓ confidence ANX vs HC	+

This table shows a summary of all studies assessing confidence in the different psychiatric disorders included in this review. In the various subparts, studies using the following populations are described: (A) subclinical OCD, (B) clinical OCD, (C) subclinical schizophrenia, (D) clinical schizophrenia, E) subclinical addiction, (F) clinical addiction, (G) subclinical depression/anxiety, and (H) clinical depression/anxiety. The results are schematically represented with ↓ signaling a significant decrease, ↑ significant increase and == no differences. Regarding the performance bias, the signs indicate the following: ++ : Study used bias free measures such as meta-d’ and/or actively kept performance equal between groups (e.g. by using a staircase procedure), +: The assessed groups had equal levels of performance, **−**: Study did not use bias free measures and did not control for performance differences between groups, or did not report accuracy measures. For more information about the most frequently used tasks, see Table 1

*HC* healthy controls, *OC* obsessive-compulsive, *OCD* obsessive-compulsive disorder, *AD* anxious-depressive, *CIT* compulsive behavior and intrusive thought, *PD* panic disorder, *SCZ* schizophrenia, *FEP* first-episode psychosis, *FOK* feeling of knowing, *GD* gambling disorder, *AUD* alcohol use disorder, *MMP* methadone maintenance patients, *CUD* cocaine use disorder, *ANX* anxiety disorder, *MDD* major depressive disorder, *S1* study 1, *S2* study 2

^a^This study has taken into account moderators (i.e. OCD-relevant contexts, responsibility level or subjective competence)

^b^This study used a prospective confidence measure

Another way of investigating the relationship between confidence and OCD features (such as repetitive checking, cleaning or doubt) is by testing the effect of manipulating confidence on OCD features or vice versa. In particular, this has been done for confidence in memory (i.e. “metamemory”). Van den Hout & Kindt^31^ were the first to show that OCD-like checking behavior leads to a decline in memory confidence levels in OCD-relevant scenarios (e.g. involving cleaning or checking), while memory performance was unaffected. Multiple studies have replicated these findings since, both for real life scenarios and mental checks^32–36^. Following the same hypothesis, another study using a repeated cleaning procedure found that memory confidence significantly increases over time for control items, yet remains stable for repeatedly cleaned items, while memory performance was equal for both items^37^. Instead of examining the effect of compulsive behavior on memory confidence, Cuttler et al.^38^ studied the effect of manipulating memory confidence on compulsive behavior and found that participants whose memory confidence is diminished, experience a higher level of doubt and more urges to check in a prospective memory task. Moreover, using the same false bio-feedback task as Lazarov et al.^26^, Zhang et al.^27^ found that the group with experimentally undermined confidence was more susceptible to distortions of confidence due to a higher reliance on the false feedback compared with the control group.

In sum, there is substantial evidence that engaging in OC behaviors lowers memory confidence, and that decreasing confidence can increase OC tendencies, supporting the idea of a link between low confidence and subclinical OC tendencies, specifically in OCD-relevant situations^31–38^. Moreover, there are multiple indications of confidence abnormalities associated with subclinical OC tendencies in the cognitive domains of interoception and perception^26,27^, such as a decrease in metacognitive efficiency^29^, although this is not supported by all studies^28,30^. These contradictory results cannot be further clarified by performance confounds, since all studies showed equal performance levels between groups. Concluding, subclinical OC tendencies are mostly associated with a decrease in confidence or metacognitive efficiency, both in OCD-relevant contexts as well as neutral task environments.

#### Clinical OCD

Of the 23 studies investigating confidence in OCD patients, most have focused on metamemory tasks (Table 2b). The pioneering study by Mcnally & Kohlbeck^39^ showed that OCD patients express lower confidence than healthy participants, whereas memory performance was equal between groups. Many studies have since replicated these findings, using both OCD-relevant and neutral tasks or stimuli^40–44^. Two studies reported that the low confidence observed in OCD patients was associated with a decrease in memory performance^45,46^. Although memory performance deficits might have been the driving force behind some reported confidence deficits (Fig. 2), many studies still find an impaired confidence in OCD patients in the absence of memory deficits^41–44^. This association does not consistently replicate, however^47–52^. To explain these contradictory results, it has been suggested that the metamemory problems in OCD are amplified by contextual factors such as a heightened subjective feeling of responsibility^53–55^. Furthermore, declining confidence levels with repetition of checks have been found in clinical OCD populations, also when controlling for anxiety levels, linking reduced memory confidence to typical OCD checking behavior^53,56^.

Declines in confidence in OCD patients have also been found in tasks evaluating perception and action^57^, general knowledge^58,59^, and interoception^60^. A recent study found no differences in the dynamic course of confidence between OCD and healthy controls in a volatile reinforcement-learning task, but did show a dissociation between confidence and action in OCD patients^61^. However, the authors did not analyze group differences for confidence precision or confidence calibration.

**Fig. 3:**
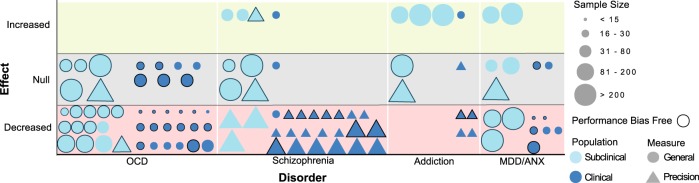
Overall confidence abnormalities in (sub)clinical psychiatry. This figure shows the overall abnormalities in confidence processes in different (sub)clinical psychiatric disorders (versus healthy controls in clinical patient groups). Every study is represented by one data point (circle or triangle). When a study existed of multiple experiments testing different populations, multiple data points were used. For all clinical studies, the sample size of the patient group is displayed. Different colors are used for subclinical (light blue) and clinical (dark blue) populations. Different symbols represent increases (on upper line) no change (middle line) or decreases (lower line) of general confidence level (circles) or precision of confidence estimation (triangles). Studies that controlled for performance biases, be it by using the bias-free meta-d’ framework, or by showing (or actively keeping) equal performance levels between groups, are outlined. For studies investigating schizophrenia that found both an increase in confidence for errors as well as a decrease in discrimination, the latter effect is displayed in this figure. The subclinical study by Rouault et al.^30^ is included in all four disorder categories. For explanation of the different confidence measures, see Fig. 1. OCD obsessive-compulsive disorder, MDD/ANX depression/anxiety disorders

Overall, most evidence points to a decrease in confidence in OCD patients in multiple cognitive domains (i.e. memory, perception, and interoception)^39–46,57–60^. This has been linked to checking behavior^53,56^, where repetitions of actions are associated with a greater distortion of confidence levels. It is, however, not fully established whether decreases in confidence, in addition to OCD-relevant situations, also extend to neutral situations. Conflicting evidence exists, such that some studies did find decreases in confidence in OCD patients using neutral tasks^40,42,43,45,58–60^, whereas other did not^48–52^. None of these studies actively controlled for performance differences between groups, but most studies did nevertheless show equal levels of performance between groups. Importantly, confidence abnormalities are likely dependent on contextual factors, since multiple studies have reported decreases in confidence in OCD patients in OCD-relevant scenarios, or specifically when patients experience heightened responsibility^47,53–57^. To our knowledge, no studies have yet investigated abnormalities in metacognitive sensitivity or efficiency in clinical OCD populations. To conclude, decreases in confidence have been found in OCD for various cognitive domains within both neutral and OCD-relevant contexts (Fig. 3). However, some studies did not find differences within the OCD population.

### Schizophrenia

Schizophrenia is a psychiatric disorder defined by positive symptoms, including hallucinations and delusions, and negative symptoms, comprising flattening of affect, loss of pleasure and social withdrawal^62^. Next to these symptoms, schizophrenic patients suffer from cognitive impairment^63^.

#### Subclinical: non-psychotic help-seeking individuals and delusion proneness

Most patients experience a prodromal phase in which symptoms gradually develop into schizophrenia or psychosis^62^. One of the predictors of transition into psychosis is cognitive impairment, with high-risk individuals exhibiting moderate to severe deficits in cognitive abilities^64^. Next to the cognitive deficits, metacognition also seems to be impaired in schizophrenia; however, the nature of the impairment is not yet fully understood.

Eight studies investigating the link between confidence and subclinical schizophrenia were identified (Table 2c). Two studies evaluated confidence in verbal memory, executive functioning, and social functioning tasks as possible neuropsychological markers in early pre-psychotic stages of schizophrenia in help-seeking adolescents.^65,66^. Scheyer et al.^66^ found no differences in either cognitive or metacognitive abilities between individuals with high versus low risk for future psychosis; yet, confidence was a significant predictor for psychosocial functioning above and beyond cognitive abilities alone. Koren et al.^65^ assessed the relationship between confidence and self-disturbance in help-seeking adolescents with or without attenuated psychotic syndrome (APS), which is considered a prodromal phase of schizophrenia. Self-disturbance is a risk factor for developing psychosis, defined as the disruption of the sense of being a self-present subject of experience and action^67^. Results showed that confidence monitoring (i.e. the correlation between confidence and actual performance) had a significant positive relationship with self-disturbance, beyond neurocognitive functioning and APS symptoms alone. This indicates that a higher level of self-disturbance was related to increased metacognitive abilities.

Regarding delusion proneness, three studies using false memory and reasoning tasks found that delusion prone subjects are more overconfident^68,69^, especially in errors^70^. Likewise, individuals with a high level of paranoia exhibited lower confidence discrimination in a visual task^71^. The authors argue that overconfidence in errors is induced by “liberal acceptance”, when partial information is deemed sufficient for having high confidence in a decision^72^. In turn, this liberal acceptance of false memories or unlikely events may promote delusions and paranoid ideation. Another study, using a general knowledge task, confirmed overconfidence in errors in individuals with high paranoia levels, but also showed that it was dependent on subjective competence and perceived difficulty^73^. They found that overconfidence in errors is exaggerated when subjects feel highly competent or deemed the question easy. However, a recent study using a perceptual task did not find any direct relationships between self-reported schizotypy symptoms and confidence level or metacognitive efficiency^30^.

In sum, prior subclinical studies have produced mixed results. One study reports no differences between high and low risk groups^66^, and one even shows improvement of metacognitive abilities with higher schizotypal symptoms^65^. Nevertheless, most of the studies, which were the most extensive in terms of participants, reported that delusion prone or highly paranoid individuals showed an overconfidence effect for errors, resulting in a diminished confidence discrimination within various cognitive domains (i.e. memory, perception and reasoning)^68–71^. Of note, a recent study indicates that this effect might also be moderated by subjective level of competence^73^. None of the studies actively controlled for performance differences.

#### Clinical Schizophrenia

Similar to research in OCD, the most considerable evidence for confidence abnormalities in schizophrenia has come from metamemory studies. Most of the 23 identified studies have either performed a source-monitoring or a false memory task (Table 2d). The majority reports that schizophrenia patients exhibit higher confidence for incorrect answers, resulting in a confidence discrimination deficit^74–83^. Schizophrenia, OCD, and post-traumatic stress disorder (PTSD) patients all exhibited lower memory performance than healthy controls, but schizophrenia patients showed a specific impairment in discrimination compared with both OCD and PTSD control groups, due to a higher confidence in errors^84^. Moritz, Woodward & Chen^85^ used the source-monitoring paradigm (Table 1) to study the developmental trajectory of confidence problems in first-episode psychosis patients (FEP). They found a confidence discrimination deficit in the FEP group due to overconfidence in errors. These results were replicated more recently in both FEP patients and high risk groups using a source-monitoring and false memory task^86,87^. Together, these findings reinforce the notion that an overconfidence in errors may serve as a risk factor for developing schizophrenia.

The inflated confidence in errors, in the absence of performance differences, was also reported in other cognitive domains, such as emotion perception^88–90^. In the perceptual domain, at similar levels of performance, schizophrenia patients showed inflated confidence in errors compared with both a healthy and an OCD control group^91^. Moreover, the amount of high confident errors significantly correlated with self-rated levels of current paranoia. Similarly, Davies et al.^92^ found that FEP patients have a significantly lower metacognitive sensitivity (*meta-d’*) compared with healthy subjects, despite similar performance and confidence levels, suggesting that schizophrenia patients are impaired in discriminating between correct and incorrect trials with their confidence judgments. However, two studies did not find such a discrimination impairment, although one did report decreased metacognitive performance in schizophrenia patients^93^. The other reported higher confidence levels in errors for healthy controls, and more high confident source misattributions in schizophrenia patients^94^.

Lastly, a study using a FOK task paired with confidence judgments found no differences in confidence level between schizophrenia patients and healthy subjects, while FOK judgments were lower in the patient group^95^. This finding was replicated using a memory task^96^.

In sum, the most consistent finding in schizophrenia patients is an inflated retrospective confidence in errors resulting in reduced confidence discrimination within multiple cognitive domains (i.e. memory, visual and emotional perception) (Fig. 3)^72,74–83,88–90^. This reduced discrimination may be attributed to a deficit in metacognitive sensitivity^92^. Furthermore, these abnormal confidence levels are already found, albeit less consistently, in early stages of the disorder (i.e. at risk populations and FEP patients)^85–87^. Concluding, schizophrenia patients show abnormal confidence discriminatory abilities induced by overconfidence in errors.

### Addiction

Addictions can be roughly divided in two categories: dependency to a substance (i.e. substance-use dependency; SUD) or to an activity (such as gambling disorder; GD). Addictions are characterized by persistent drug use or maladaptive behavior despite negative consequences^97^. SUDs and behavioral addictions have a common underlying neural mechanism that governs the development and sustenance of these disorders^98^. Next to classic symptoms of habit forming and craving, addicted individuals are also impaired in a broad spectrum of cognitive functions^99^.

#### Subclinical addiction

Three studies investigating confidence in subclinical addiction were identified (Table 2e). Two studies divided a student population into probable pathological gamblers, problem gamblers and no-problem gamblers and used a general knowledge task^100,101^. Goodie^100^ found that pathological gamblers have significantly higher confidence, but also lower task performance, compared with the other groups, resulting in higher overconfidence. Similarly, Lakey et al.^101^ showed that non-problem gamblers were less overconfident than the other two groups, with no differences between the pathological and problem gamblers. Both studies also found a significant positive correlation between gambling severity and overconfidence. Considering SUD, a recent study using a perceptual task found no direct relationship between self-reported alcoholism symptoms and either confidence level or metacognitive efficiency in the general population^30^.

Taken together, these few studies showed some evidence for confidence abnormalities in subclinical GD within the semantic memory domain, pointing to increased overconfidence in a general context^100,101^ (Fig. 3). However, task performance was not held equal between groups, rendering it difficult to draw firm conclusions. Furthermore, these findings did not extend to links between alcoholism symptoms and confidence within the perceptual domain^30^. The link between confidence abnormalities and subclinical symptoms of addiction is therefore not yet apparent.

#### Clinical addiction

A total of five studies have investigated confidence in addiction (Table 2f). One study assessed confidence in GD patients and healthy controls using a non-gambling grammar task and reported similar confidence levels in both groups, while GD patients exhibit lower performance^102^. However, confidence correlated with performance in healthy controls, but not in GD patients, suggesting an abnormal confidence processing in gamblers. Considering SUD, Le Berre et al.^103^ studied confidence in alcohol-use disorder patients using a memory task with a prospective FOK measure. Results showed that alcohol use disorder patients had a significantly worse memory performance than healthy controls, and were less accurate regarding their FOK judgments as they overestimated their recognition performance. Moreover, a significant positive correlation was found between memory deficits, executive dysfunction and metamemory impairment in alcohol use disorder patients. In another study, using a visuo-perceptual task in which performance was held constant, active cocaine addicted individuals displayed a decreased metacognitive efficiency compared with remitted cocaine users and healthy subjects^104^. Interestingly, the remitted group did not differ from the healthy controls. Both cocaine user groups did not differ with regards to peak drug usage, suggesting that the results cannot be attributed to a greater lifetime addiction severity in active users.

To date, two studies have examined confidence in a population of opiate dependent patients receiving methadone maintenance treatment. Mintzer & Stitzer^105^ found that patients reported significantly higher confidence for incorrect choices in a memory task compared with healthy subjects, resulting in worse confidence discrimination. Recently, Sadeghi et al.^106^ found lower metacognitive efficiency for patients using a perceptual task, while no differences in mean confidence levels or performance could be detected. In the memory domain, however, patients exhibited lower performance but similar metacognitive efficiency than controls. These findings suggest that separate metacognitive systems might exist for different cognitive domains.

Summing up, a single study in GD patients showed a disconnection between confidence and accuracy, indicating a deficiency in metacognition^102^. Replications using bias free measures of confidence are needed in order to confirm this effect. In SUD patients, multiple studies correcting for performance differences and using bias-free confidence measures reported inflated retrospective confidence for errors and thus decreased confidence discrimination, as well as diminished metacognitive efficiency. This abnormality was found in both memory and perceptual domains^105,106^, and improved in remitted patients^104^. Replications and direct comparisons between addiction subtypes are needed to confirm the generalizability of these findings. Concluding, multiple bias-free studies reported a decrease in confidence discrimination and metacognitive efficiency in SUD patients (Fig. 3). However, for GD patients, more research is needed.

### Anxiety and depression

Major depressive disorder (MDD) and anxiety disorders are common disorders with a lifetime prevalence of 16.2% and 28.8%, respectively^107,108^. Since they are both classified as mood disorders and are highly comorbid, they are considered jointly. MDD and anxiety disorders share a negativity bias in information processing, reflecting a greater focus on negative input^109–112^. While general deficits in cognition are established symptoms in these disorders^113,114^, studies investigating confidence disorders are scarce. However, the well-known hallmarks of both disorders: negative self-concepts, rumination and indecisiveness^109^, suggest that patients show a negative confidence bias.

#### Subclinical anxiety and depression

Subclinical levels of depression and anxiety are common among the general population^115^. Five studies researching subclinical anxiety or depression were identified (Table 2g). Stone, Dodrill & Johnson^116^ used a general knowledge task in four groups from a general population sample: (1) non-depressed non-anxious, (2) non-depressed anxious, (3) depressed non-anxious, and (4) depressed anxious. They reported lower confidence levels in depressed non-anxious individuals compared with the control group (non-depressed, non-anxious), in the absence of performance differences. Surprisingly, the depressed anxious group did not differ from the control group on any measure, suggesting that the presence of anxiety itself might counterbalance the confidence abnormalities found in depression. Soderstrom, Davalos & Vázquez^117^ divided a non-clinical sample into non-, mild-, and moderate depression groups and used a memory task with a JOL measure (i.e. prospective confidence). While results showed overconfidence in all three groups, mildly depressed subjects exhibited significantly lower overconfidence than the other groups. No differences in calibration were found between the non- and moderately-depressed groups. However, caution must be taken when interpreting these results, as performance levels were significantly different between the groups. The authors of a third study divided a large group of undergraduates into depressed and non-depressed groups and asked participants to predict future events^118^. They reported overconfidence in the depressed group, but this was fully driven by differences in prediction performance: while reporting similar levels of confidence, depressed individuals showed a decreased performance in predicting future events compared with the non-depressed group. Moreover, the lack of confidence differences between groups could be explained by the use of valenced life events rather than a neutral task: since depressed subjects commonly have a negative self-concept and a general focus on negative events^109^, they may have a high confidence that negative events could happen.

One study did not detect any association between depression and/or anxiety symptoms and various confidence measures obtained via several cognitive tasks assessing executive functioning, memory and social emotional functioning^119^. However, Rouault et al.^30^ did find a significant negative relationship between self-reported depression and anxiety symptoms and confidence level in the general population, indicating that individuals with higher depression or anxiety symptom scores report lower levels of confidence.

Together, the research on metacognition in mood disorders remains inconclusive to date due to contradictory results. Two studies reported underconfidence in the subclinical depressed groups within perceptual and semantic memory domains;^30,116^ two studies showed overconfidence due to performance deficits^117,118^ using prediction and memory tasks, and one study reported null findings in various cognitive domains (i.e. executive functioning, memory and emotional processing)^119^. Moreover, individuals with both depression and anxiety symptoms did not show confidence abnormalities. However, some of these studies were confounded by differences in performance, which could have caused false reports of overconfidence. Regarding only the studies that did correct for performance differences and used retrospective confidence judgments^30,116^, all reported an effect of underconfidence.

#### Clinical anxiety and depression

In MDD patients, four studies were identified that mostly reported underconfidence compared with healthy controls using different paradigms (Table 2h). One study found decreased confidence discrimination in both current and recovered MDD patients using a general knowledge task^120^. This effect significantly correlated with depression severity, such that patients with more severe depression showed lower confidence levels and discrimination. A second study using four different decision tasks (i.e. an episodic memory, general knowledge, perceptual discrimination and a social judgment task) found that MDD patients reported lower confidence levels than the control group, whereas recovered patients did not^121^. In both studies, performance was equal between the groups. In a third study, MDD patients exhibited lower performance in a memory task than a control and a chronic-fatigue syndrome patient group. This was accompanied by greater underconfidence in the MDD group, both when judgments were made after every single trial and after a block of trials^122^. Lastly, a recent study using an emotional perception task found no interaction between group and confidence in a model explaining incorrect responses^123^. However, in line with previous findings, the authors did find a significant association between low confidence levels and high depression severity scores.

To our knowledge, there are no studies to date examining confidence focusing solely on anxiety patients versus healthy controls. However, a few studies investigating OCD used anxiety disorder patients as a clinical control group. Two studies found no difference between anxiety or panic disorder patients and healthy controls regarding confidence^59,60^, whereas another study showed that anxious controls had lower confidence levels^56^. A recent study, which did not include a healthy control group, found that anxious and OCD patients had similar levels of memory confidence^47^.

In summary, most studies showed a reduction of confidence levels in MDD in different cognitive domains (i.e. memory, visual and social perception)^120–122^. Furthermore, some studies showed greater levels of underconfidence for current versus recovered MDD patients^121^, whereas other studies did not report any differences^123^. Mixed results were found for anxiety disorders: two studies showed decreased confidence levels similar to OCD when compared to healthy controls within the memory domain^47,56^, whereas two other studies did not find such differences using general knowledge and interoception paradigms^59,60^. Concluding, depression patients mostly showed an effect of underconfidence, whereas this effect was not clear-cut for anxiety patients (Fig. 3).

### Transdiagnostic psychiatry

Transdiagnostic psychiatry is an emerging scientific field which attempts to decipher the cognitive, affective and neurobiological processes underlying complex behavior by relating them to symptom dimensions. Since this approach transcends traditional diagnostic categories, it has the potential to refine the current nosology-based clinical classifications beyond the classical Diagnostic and Statistical Manual of Mental Disorders (DSM) diagnostic criteria^124,125^. The underlying idea of this approach is that cognitive and brain-related functions (e.g. those relating to confidence processing) might map more closely onto symptomatology than DSM diagnoses.

A recent study by Rouault et al.^30^ leveraged such a transdiagnostic psychiatry approach to investigate the relationship between confidence and psychiatric symptomatology in the general population. A large sample from the general population performed a perceptual decision-making task and answered self-report questionnaires spanning a range of psychiatric symptoms, including depression, general anxiety, schizotypy, impulsivity, OCD, social anxiety, eating disorders, apathy and alcohol dependency (Experiment 1: *n* = 498. Experiment 2: *n* = 497. See Table 2a, c, e, g). The relationships between accuracy, decision parameters, confidence and metacognitive efficiency (*meta-d’/d’*) were examined. Results showed that the symptoms were not associated with decision parameters, but that higher levels of depression and anxiety symptoms were significantly associated with decreased confidence. Furthermore, a factor analysis was carried out to retrieve a parsimonious latent structure that best explained the variance at the item level of all questionnaires, which identified three symptom dimensions: Anxious-Depression (AD), Compulsive Behavior and Intrusive Thought (CIT) and Social Withdrawal (SW). The AD dimension was significantly associated with lower confidence and higher metacognitive efficiency, whereas the CIT cluster was related to higher confidence and a lower metacognitive efficiency. The metacognitive efficiency results did, however, not survive correction for multiple comparisons and must be interpreted with caution. Lastly, none of the three symptom dimensions showed a relationship with decision parameters, indicating that psychiatric symptoms are related to shifts in confidence, but not in performance. Therefore, changes in confidence may represent a specific behavioral correlate of subclinical psychopathology that could be an important component of transdiagnostic psychiatry.

## Discussion

In this review we sought to obtain an answer to the question whether confidence judgments are abnormal across psychiatric disorders. We found evidence for confidence abnormalities across a variety of psychiatric disorders, which take specific directions for the different populations (Fig. 3). For (sub)clinical OCD, the most consistent finding is a decrease in confidence level, especially related to typical OCD contexts, such as checking behavior. Regarding (sub)clinical schizophrenia, we primarily found increased confidence in errors resulting in a decrease of discrimination and metacognitive sensitivity. This diminished discriminatory ability between correct (real) and incorrect (imagined) situations fits core schizophrenia symptoms such as delusions and hallucinations, and was recently also found to be dependent on subjective competence. In clinical addiction, an increase in confidence—leading to a decrease in confidence discrimination and metacognitive efficiency—was found, which corresponds to the symptomatic lack of self-insight in this population^126^. Subclinical addiction has not been studied as extensively, but overconfidence was found in subclinical GD. In clinical anxiety and depression, reductions in confidence levels were found, which fit with the negative information processing bias observed in mood disorders^110^. However, subclinical studies show mixed results and no studies using anxiety patients as the primary group of interest have been performed to date. Together, these results demonstrate that clinical and subclinical studies generally show similar results.

While these results suggest that there are abnormalities in confidence estimations in psychiatric patients, another important question is how these abnormalities relate to psychiatric disorders. Are these abnormalities closely linked or even underlying psychiatric symptoms? Are they a result of the disorder or perhaps only a byproduct without any significance for symptomatology? The studies discussed in this review indicate that there is a close interplay between psychiatric symptoms and confidence. For instance, several studies found that abnormal levels of confidence are already present in non-clinical populations with psychiatric tendencies or subclinical prodromal populations^26,27,29,85–87,92^. Moreover, a normalization of confidence abnormalities was found in three studies after patients recovered^104,120,121^. Furthermore, four studies found direct correlations between confidence abnormalities and symptom severity^100,101,120,123^. The interaction between psychiatric symptoms and confidence abnormalities was also demonstrated by studies showing that engaging in compulsive behaviors lowered confidence levels, whereas undermining confidence lead to increases in compulsive tendencies^31–38^, indicating that confidence and pathological behavior are coupled. While the evidence for the strong relationship between confidence and psychiatric symptoms is convincing, the directionality of the effect is not unequivocal and should therefore be further explored in future studies using causal manipulations of confidence or longitudinal designs.

These findings raise many questions and give way to research advancing our understanding of confidence abnormalities in psychiatry. Confidence is not a unitary construct, since confidence abnormalities are differently expressed in various contexts^54,73^, and the role of context in confidence abnormalities should be further identified. For example, it is possible that confidence abnormalities aggravate in a symptom-related context. For instance, a gambler might be overconfident in general, but show an even increased overconfidence during gambling. Another interesting future avenue would be to study if normalization of confidence deviations would translate into decreased symptom severity, and vice versa. Interestingly, a recent paper showed that adaptive training can cause a domain-general enhancement of metacognitive abilities in the general population^127^. Up to now, several forms of metacognitive training have been developed as treatment for psychiatric patients. Importantly, recent meta-analyses indicated that they were effective in reducing symptoms within a wide range of psychiatric disorders^128,129^. Furthermore, metacognitive training, as well as antipsychotic medication, have been shown to attenuate overconfidence in errors in schizophrenia patients^130,131^. Future work should focus on translating current knowledge about confidence abnormalities in psychiatry to new treatment interventions, tailored to specific confidence abnormalities. Furthermore, it remains uncertain whether confidence abnormalities in psychiatry generalize over different cognitive domains and contexts. Few studies have systematically and directly studied the transfer of confidence abnormalities across different domains within a population and showed mixed results favoring either domain-general^57^ or domain-specific^106,121^ views. However, the majority of the discussed studies used metamemory tasks; therefore, more research is needed to establish the generalizability of confidence disruptions to other cognitive domains. More knowledge about the relationship between confidence abnormalities in various domains and psychiatric disorders may eventually allow for personalized therapies focusing on individual deficits.

Next to using the traditional DSM diagnostic categories, it is important to study confidence using a transdiagnostic approach focusing on the level of symptoms. Recently, Rouault et al.^30^ used a transdiagnostic approach and found that a symptom cluster of compulsivity and intrusive thoughts is related to heightened confidence, whereas an anxiety and depression cluster is related to lowered confidence in a large sample of the general population. Importantly, their results were less pronounced when symptoms were related to confidence abnormalities in the traditional diagnostic categorical (i.e. disorder-specific) way. This may indicate that confidence abnormalities are better explained by specific symptom clusters than disorder categories that are heterogeneous in their display of symptoms, because they show overlap with other disorders. For example, there might be large individual variety in the role that anxiety^132^ and compulsivity play in psychiatric disorders such as addictions and OCD, resulting in different propensities for under- or overconfidence. Currently, it is not clear if and how these transdiagnostic findings generalize to clinical groups, although our findings seem to suggest that confidence abnormalities are similar between clinical and subclinical populations. An interesting avenue for future work is to apply transdiagnostic approaches to clinical groups and investigate whether symptom-based classification improves correlations with confidence abnormalities compared to classical DSM-based classification. Moreover, in addition to the data-driven transdiagnostic techniques adopted by Rouault et al.^30^, other theory-driven techniques fitting the Research Domain Criteria (RDoC) framework should be used to further explore confidence abnormalities in psychiatric populations^133^. Bearing in mind the advantages of the transdiagnostic approach, new treatment interventions focusing on treatment of confidence abnormalities related to specific symptom clusters instead of DSM classifications could be a promising new avenue. Furthermore, next to confidence being an important transdiagnostic factor associated with psychiatric disorders, many other factors have been shown to be of transdiagnostic value, such as neurocognitive deficits and motivation^134–136^. These factors may also contribute to confidence deviations within psychiatric populations^75^, which makes for an important area of future research.

Confidence can be viewed as a broader concept than the cognitive operationalization reviewed in this paper, relating to themes relevant to psychiatry such as trust and self-confidence^137,138^. In order to gain a wider perspective on the role of confidence in psychiatry it would be interesting to explore how these themes are related and investigate the phenomenology of confidence abnormalities in these disorders.

The reviewed studies also indicate that there are methodological shortcomings in the field. Most of the reported studies suffered from (one of) two limitations. First, they did not account for performance differences between groups of interest. Performing better at a task leads to an increase in confidence^23^, and there is growing evidence that confidence judgments guide future behavior^139^. It is thus crucial to control for performance differences to isolate effects in confidence. Second, they did not use bias free measures next to the conventional measures of confidence level, such as calibration and discrimination. Bias free measures account for performance differences and response biases and provide more in-depth information about one’s metacognitive abilities. Future work would benefit from using tasks that control for potential performance differences and use bias free measures such as *meta-d’* (although these measures require a considerable amount of trials to obtain sufficient statistical power^8^). Furthermore, a discrepancy exists in how confidence is assessed inside and outside the clinical fields, with more effort toward a normative definition of confidence^1^, operationalization using (Bayesian) computational frameworks^139,140^ and confidence evaluation, incentivization or assessment^141^ outside of clinical fields. Adopting these standards in clinical research could help improving our knowledge about confidence abnormalities in psychiatry. Lastly, there is more and more research into the neurobiological basis of confidence, which shows that brain areas such as the lateral and medial prefrontal cortex and insula are related to confidence encoding^142^. Interestingly, these brain areas also play a central role in the various psychiatric disorders discussed in this review^112,143–146^. Therefore, studying the neural mechanisms responsible for the confidence abnormalities observed in these populations is an important future research endeavor.
